# Methuosis, Alkaliptosis, and Oxeiptosis and Their Significance in Anticancer Therapy

**DOI:** 10.3390/cells13242095

**Published:** 2024-12-18

**Authors:** Elżbieta Bartoszewska, Kamila Florek, Karol Zagórski, Martyna Gachowska, Anna Wietrzyk, Agata Hutny, Agnieszka Nowakowska-Toporowska, Julita Kulbacka

**Affiliations:** 1 Faculty of Medicine, Wroclaw Medical University, L. Pasteura 1, 50-367 Wroclaw, Poland; elzbieta.bartoszewska@student.umw.edu.pl (E.B.); kamila.florek@student.umw.edu.pl (K.F.); karol.zagorski@student.umw.edu.pl (K.Z.); martyna.gachowska@student.umw.edu.pl (M.G.); anna.wietrzyk@student.umw.edu.pl (A.W.); agata.hutny@student.umw.edu.pl (A.H.); 2 Student Research Group No. K148, Faculty of Pharmacy, Wroclaw Medical University, Borowska 211A, 50-556 Wroclaw, Poland; 3 Department of Prosthodontics, Wroclaw Medical University, Krakowska 26, 50-425 Wroclaw, Poland; agnieszka.nowakowska-toporowska@umw.edu.pl; 4 Department of Molecular and Cellular Biology, Faculty of Pharmacy, Wroclaw Medical University, Borowska 211A, 50-556 Wroclaw, Poland; 5 Department of Immunology and Bioelectrochemistry, State Research Institute Centre for Innovative Medicine, Santariškiu˛ g. 5, LT-08406 Vilnius, Lithuania

**Keywords:** methuosis, alkaliptosis, oxeiptosis, cancer therapy, cell death pathways

## Abstract

Understanding morphological, biochemical, and functional aspects of cell death is essential for targeting new cancer therapies. Even though many different mechanisms of cell death are identified, it is crucial to highlight the role of new and lesser-known pathways, including methuosis, alkaliptosis, and oxeiptosis. The aim of this review was to summarize the data about cell death mechanisms—methuosis, alkaliptosis, and oxeiptosis—and their role in cancer treatment. Unique molecular mechanisms and cellular outcomes characterize each of these forms of cell death. This research on methuosis, alkaliptosis, and oxeiptosis provides a better understating of cell death biology and creates novel opportunities for neoplasm management.

## 1. Introduction

### 1.1. General Overview of Cell Death Mechanisms

Understanding the diverse mechanisms of cell death is crucial in the context of cancer therapy. The definitions and classification of cell death based on morphological, biochemical, and functional characteristics are continually being updated. The most recent 2023 guidelines refine the classification of regulated cell death (RCD) types to include apoptosis (type I), autophagy-dependent cell death (type II), and regulated necrosis (type III). Furthermore, the updated classification incorporates emerging evidence on other forms of RCD, such as necroptosis, NETosis [1,2], pyronecrosis [3,4], pyroptosis [5], ferroptosis, and parthanatos, which are defined by distinct molecular mechanism and biochemical pathways [6,7,8].

RCD is controlled by multiple interconnected signaling pathways and molecular mechanisms, and it plays essential roles in processes like organogenesis, tissue remodeling, elimination of unnecessary or damaged cells, and regulation of cell populations and their functions. RCD can also be induced by external stressors, such as drugs and cancer therapies, which disrupt the cellular microenvironment [7,9].

### 1.2. Methuosis, Alkaliptosis, and Oxeiptosis

The following sections of the paper will explicate three selected forms of cell death—methuosis [10], alkaliptosis [11,12], and oxeiptosis [13,14]—which are relatively newly discovered and lesser known, but potentially significant mechanisms in the context of cancer therapy.

Methuosis is a nonapoptotic form of cell death characterized by the vacuolization of the cytoplasm and a reduction in cell size. This process is distinct from apoptosis and necrosis, and it involves the disruption of macropinocytosis, leading to excessive accumulation of vacuoles. It has been observed in various cancer cell lines, suggesting its potential as a target for cancer therapy [10].

Alkaliptosis, another RCD discovered in 2018, is a form of regulated cell death distinct from apoptosis, necroptosis, and other well-known cell death mechanisms. This process is characterized by its dependency on intracellular alkalization, which is a unique feature compared to other forms of cell death. Alkaliptosis involves the swelling of cellular organelles and the rupture of the plasma membrane, leading to cell death [11,12].

Oxeiptosis is a distinct cellular pathway that has emerged as a unique form of cell death. It is characterized by its reliance on intracellular reactive oxygen species (ROS) sensing and activation. This process is specifically triggered by elevated levels of ROS [13]. Oxeiptosis involves the engagement of KEAP1, a well-studied intracellular ROS sensor, with the mitochondria-tethered phosphatase PGAM5. This pathway leads to a non-inflammatory, caspase-independent, and apoptosis-like cell death phenotype. The role of oxeiptosis extends beyond virus infections, potentially influencing diverse biological functions and developmental stages. It is also speculated to play a role in tumorigenic processes and neurological development. This pathway represents an integrated mechanism that unifies cytoprotective and pro-apoptotic responses to oxidative stress, mitigating damage caused by ROS [13,14].

### 1.3. Significance in Cancer Therapy

Recent advances in understanding cell death mechanisms have opened new avenues in cancer therapy. Specifically, the roles of methuosis, alkaliptosis, and oxeiptosis have gained significant attention for their potential in treating various types of cancer. Methuosis has emerged as a novel approach, especially in the context of triple-negative breast cancer (TNBC), which currently lacks effective targeted therapeutic agents. The unique vacuole-presenting modality of methuosis promotes tumor cell death, offering a new strategy to overcome the limitations of existing treatments [15]. A growing amount of research demonstrates the potential of small-molecule compounds in specifically inducing methuosis in tumor cells, with minimal effects on normal cells. This specificity suggests that targeting tumor cell methuosis could be an effective prevention strategy for cancer [16]. Moreover, recent discoveries of azaindole-based compounds that cause vacuolization in cancer cells underscore the potential of methuosis inducers as novel therapeutics [17].

Alkaliptosis, a pH-dependent form of regulated cell death, has been identified as a promising strategy for cancer therapy across multiple tumor types, particularly pancreatic cancer [12]. The process is triggered by small-molecule compounds, such as JTC801, and involves major signaling pathways like the NF-κB-CA9 pathway and the ATP6V0D1-STAT3 pathway [11]. The potential of alkaliptosis in tumor therapy is underscored by its unique molecular mechanisms and regulatory networks, which are yet to be fully understood [18]. Notably, the combination of compounds such as arenobufagin with traditional chemotherapeutics has shown synergistic effects in inhibiting tumor growth by inducing alkaliptosis, suggesting new combination therapies for cancer treatment, including gastric cancer [19].

Oxeiptosis represents a novel tumor-suppressive mechanism that has shown effectiveness in reducing tumor growth in models like the HT-29 xenograft mouse model [20]. This ROS-sensitive, caspase-independent, non-inflammatory cell death pathway is crucial for protection against inflammation induced by ROS or ROS-generating agents, such as viral pathogens [13]. Studies on compounds such as alantolactone, which promotes ROS generation exclusively in cancer cells, further illustrate the potential of oxeiptosis in specifically targeting cancer cells, as demonstrated in ovarian cancer research [21]. The severity of oxidative damage appears to dictate the mode of cell death, linking oxidative stress, apoptosis, and oxeiptosis, with significant implications for cancer therapy [22].

Thus, the exploration of methuosis, alkaliptosis, and oxeiptosis offers promising new pathways for cancer treatment. These mechanisms provide novel approaches to specifically target tumor cells, potentially leading to more effective and less toxic cancer therapies. As research continues to unveil the intricacies of these cell death modalities, their significance in the realm of cancer therapy is likely to grow, offering hope for better treatment strategies in the fight against cancer.

## 2. Methuosis

**Figure 1:**
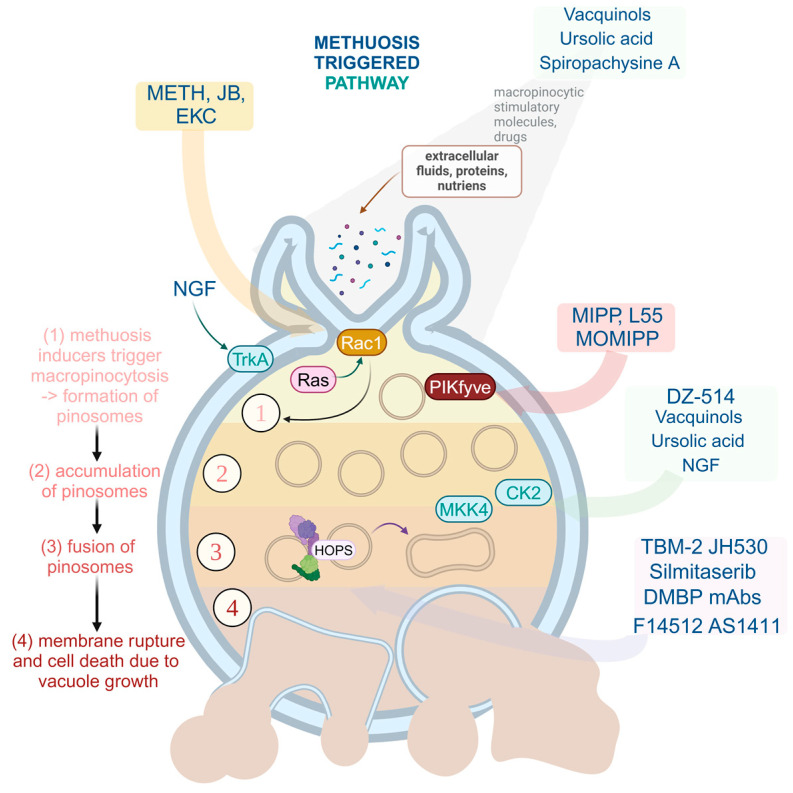
Mechanistic pathway of methuosis-induced vacuolization. The numbered regions within the cell (1–4) represent the distinct stages in the methuosis process: (1) initiation of macropinocytosis: methuosis inducers activate pathways that trigger macropinocytosis, resulting in the formation of small vesicles (pinosomes) within the cytoplasm; (2) accumulation of pinosomes: cells progressively collect these pinosomes, accumulating them in the cytoplasm; (3) vesicle fusion: the pinosomes fuse to form larger vacuoles, marking an advanced stage of methuosis; (4) membrane rupture and cell death: large vacuoles continue to grow, eventually leading to membrane rupture and cell death. NGF—nerve growth factor; EKC—epimedokoreanin C; JB—jaspine B; METH—methamphetamine; TBM-2—tubeimoside 2; DMBP—methyl 2,4-dihydroxy-3-(3-methyl-2-butenyl)-6-phenethylbenzoate; mAbs—monoclonal antibodies; TrkA—tyrosine kinase receptor A; MKK4—mitogen-activated protein kinase kinase 4; CK2—casein kinase 2; HOPS—homotypic fusion and vacuole protein sorting complex.

Methuosis is a nonapoptotic type of cell death that has been discovered in this century. The process is caused by exorbitant stimuli, which cause cytoplasmic absorption, creating tiny vesicles that eventually combine to form large vacuoles. As a result, cellular metabolic activity declines and cell membrane rupture occurs, leading to the death of cells (Figure 1) [23]. The first observation of cell demise influenced by increased vesicular fluid absorption and the formation of inflated macropinosomes was made in 2008. The connection was made by studying the effect of overstimulation of micropinocytosis on Ras-triggered death in glioblastoma cells [24]. The researchers noticed a previously unseen process that originated from cytoplasmic vacuolation, followed by cell swelling and plasma membrane rupture. After additional tests, nucleosomal DNA fragmentation, caspase activation, ATP depletion, and increase in autophagic activity were observed. This research concluded by naming the newfound mechanism “methuosis” from the Greek word *methuo*, meaning “drink to intoxication” [25].

Macropinocytosis is a starting point of methuosis. It is an actin-driven mechanism that is activated by a variety of stimuli. It begins with plasma membrane ruffling and the creation of a pinocytotic cup, which envelopes extracellular fluid and produces a pinosome by membrane merger at the tip of a lamellipodium-like formation. The developing pinosome absorbs ions, nutrients, metabolites, toxins, pharmaceuticals, and even pathogens like viruses and bacteria, suspended in extracellular fluid. Physiologically, pinosomes migrate centripetally. Their acidity increases, their diameter shrinks, and they become tubulated, which is required for sorting and distributing the vesicles and their content [10]. This mechanism is significantly disrupted in methuosis, in which the cells “drink themselves to death”. Fluid intake is increased by macropinocytosis, and instead of shrinking, the vesicles enlarge, do not acidify, stay non-functional, and do not merge with lysosomes, but rather with each other. As a consequence of the creation of immense vacuoles and fatal cell expansion, plasma membrane rupture and cell destruction follow [10,24].

**Figure 2:**
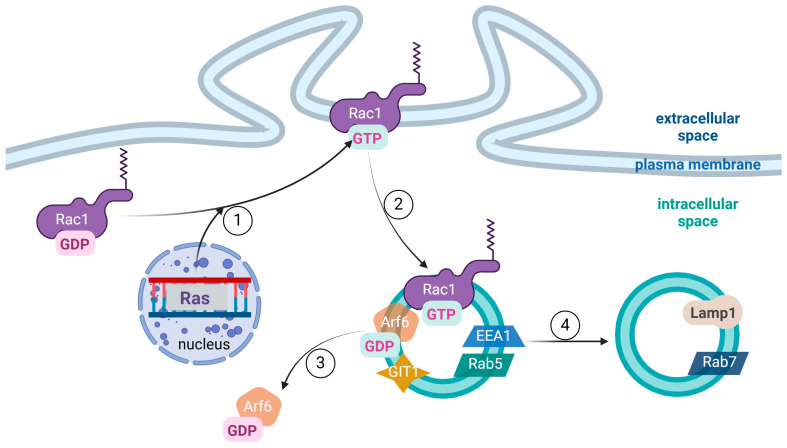
Vacuolization processes induced by Ras-related methuosis stimulants, where: (1) expression of Ras (oncogenic H-RasG12V and K-RasBG12V) activates GTPase Rac1; (2) activated Rac1 induces micropinocytosis; (3) Rac1 connects with GIT1, leading to Arf6 inactivation, preventing macropinosome recycling to the plasma membrane; (4) macropinosomes develop late endosomal characteristics and merge to create vacuoles [30]. GDP—guanosine diphosphate; GTP—guanosine triphosphate; Arf6—ADP-ribosylation factor 6; GIT1—G protein-coupled receptor kinase; EEA1—early endosome antigen 1; Lamp1—lysosome-associated membrane protein 1.

Methuosis is a nonapoptotic form of cell death characterized by Ras hyperactivation, which promotes macropinocytosis through activation of Rac1 family proteins and impedes macropinosome recycling due to the absence of Arf6-GTP (Figure 2). This pathway results in extensive cytoplasmic vacuolization rather than DNA fragmentation typical of apoptosis or membrane rupture seen in necrosis [26]. During methuosis, large vacuoles, marked by late endosomal proteins such as LAMP1 and Rab7, accumulate due to ongoing macropinocytosis and limited recycling, leading to cell death through disrupted cellular homeostasis rather than programmed (apoptotic) or necrotic pathways [27]. Specific stimulants can promote vacuolization pathways based on distinct mechanisms, which we classify as follows.

- Class I: Vacuole formation driven by Ras gene activation, which initiates a sequence of events leading to Rac1 activation. This Rac1 activation promotes macropinocytosis. Concurrently, Rac1 in its activated form interacts with G protein-coupled receptor kinase-interacting protein 1 (GIT-1), deactivating ADP-ribosylation factor 6 (Arf6) and preventing macropinosome recycling into the plasma membrane [28]. As a result, macropinosomes acquire late endosomal characteristics and accumulate, eventually fusing to form a vacuole.
- Class II: Vacuole formation induced by external agents, including casein kinase 1 (CK1), mitogen-activated protein kinase kinase 4 (MKK-4), nucleolin (Nuc), nerve growth factor (NGF), Arf6, G protein-coupled receptor kinase-interacting ArfGAP 1 (GIT1), and early endosome antigen 1 (EEA1) [24,29]. These agents initiate vacuolization through various intracellular signaling pathways independently of the Ras/Rac1 pathway [29].

Mutations of RAS genes were discovered in around 30% of human cancers, such as gastric cancer and glioblastoma [28]. The studies show that in cases where cancer causes the expression of the K-RAS gene, like osteosarcoma, the suppression of the mutation of the aforementioned gene in the physiological epithelial cells of a human provides an anti-tumor property [31]. A similar effect shows the activation of the tyrosine kinase receptor A (TrkA) in myeloma tumor cells. It promotes the existence and differentiation of typical neurons while inducing micropinocytosis in tumor cells, leading to their death. Another process leading to methuosis is the suppression of the 6-phosphofructo-2-kinase/fructose-2,6-biphosphatase 3 (PFKFB3) gene in sarcomatoid and epithelioid cells. The same result was created by suppression of PIKFYVE (a class III phosphoinositide (PI) kinase) and an increase in Rac1 mRNA and Rac1 protein expression in human nasopharyngeal carcinoma cells [26,32].

There are many substances that can induce methuosis (Table 1). Among them, synthetic indole-based chalcones (MIPP, MOMIPP) seem to have the most promise. They have been found to overactivate RAS genes, causing rapid vacuolization at distinctly low concentrations in various cancer cell cultures—among others, glioblastoma cells [27]. The usage of extracellular fluorescent yellow and time-lapse microscopy allows observation of how those substances promote the creation of macropinosomes through plasma membrane reduction. Those vesicles rapidly merge and do not participate in lysosomal fusion or membrane recirculation. Moreover, MOMIPP activates the JNK1/2 stress kinase pathway, causing the phosphorylation of PIKFYVE, which is another inducer of methuosis. It was also concluded that MIPP triggers methuosis about 10 times quicker than the Ras pathway [26]. Other compounds that trigger methuosis are also presented in Table 1.

## 3. Alkaliptosis

**Figure 3:**
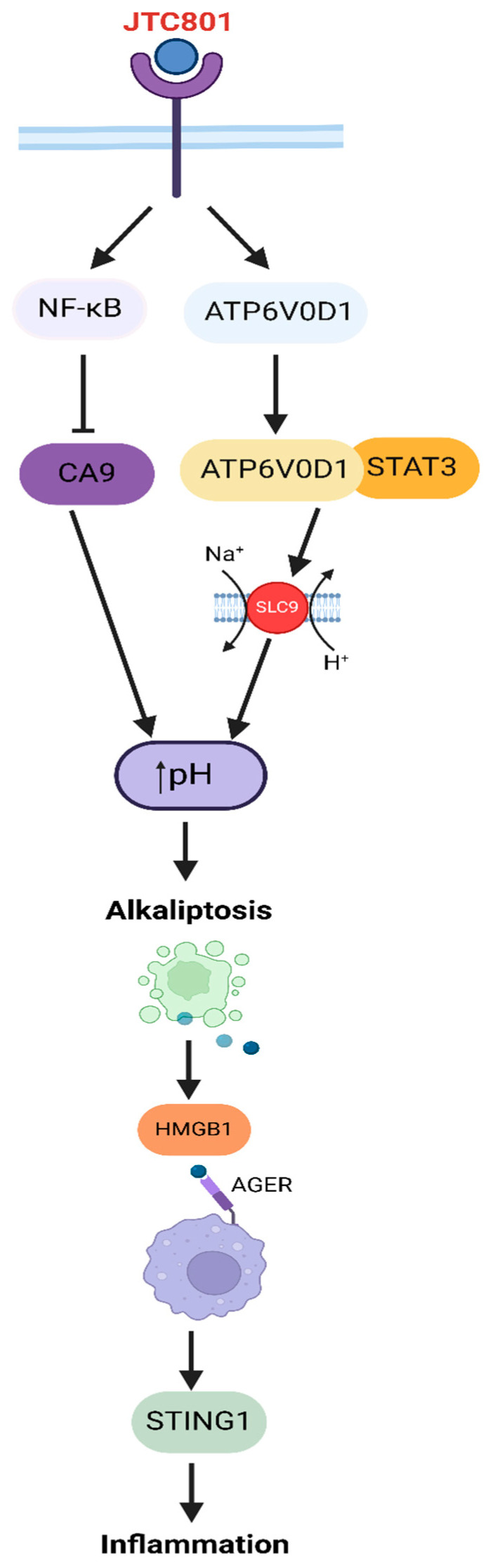
Schematic summary of alkaliptosis pathway induced by JTC801, a pH-dependent form of cell death. Upon binding to its receptor, JTC801 activates two distinct signaling branches. (1) JTC801 activates the NF-κB pathway, which subsequently upregulates carbonic anhydrase 9 (CA9). CA9 plays a role in maintaining cellular pH by modulating the acid–base balance. (2) JTC801 activates ATP6V0D1 and STAT3. ATP6V0D1 contributes to the alkalinization of the intracellular environment, further promoting the increase in cellular pH [11,43,44].

Alkaliptosis was described for the first time during a search for novel human pancreatic ductal carcinoma (PDAC) therapies in 2018. It is a regulated cancer cell death induced by GPCR opioid-related nociceptin receptor 1 (OPRL1) activation with JTC801, which leads to lethal intracellular alkalization (Figure 3) [43]. The mechanism of alkaliptosis is independent of genes inducing apoptosis, necrosis, and autophagy. Moreover, molecular markers and specific inhibitors of previously mentioned cell death types and ferroptosis are not associated with alkaliptosis [11,43].

Alkaliptosis starts with NF-κB activation by JTC801 and subsequent inhibition of carbonic anhydrase 9 gene (CA9) expression, resulting in cell pH disbalance. This leads to cytosolic alkalization and tumor growth suppression [18]. In the NF-κB pathway, phosphorylation and acetylation are critical regulatory steps in its activation. NF-κB activation is facilitated by acetylation through acyl-CoA synthetase short-chain family member 2 (ACSS2). Additionally, phosphorylation of the NF-κB inhibitor IκBα by the IKK complex (composed of IKKα, IKKβ, and IKKγ) leads to IκBα degradation. This degradation releases the NF-κB subunits nuclear factor kappa B subunit 1 (NFKB1) and RELA proto-oncogene (RELA), enabling their translocation into the nucleus, where they regulate gene expression [11].

However, there is another pathway leading to alkaliptosis, also mediated by JTC801, enhancing interaction between ATPase H^+^-transporting V0 subunit D1 (ATP6V0D1) and signal transducer and activator of transcription 3 (STAT3). ATP6V0D1 forms a complex with STAT3, which promotes lysosome acidification and cytosol alkalization [45]. ATPase H+ subunit action is associated with activation of ion transporters, mainly among solute carrier family 9 members (SLC9).

The expression of some SLC9A family members is conditioned by STAT3. There are some neoplasms resistant to drug therapies due to STAT3 overactivity, and in those cases, STAT3-dependent alkaliptosis may be an appropriate treatment strategy. Moreover, alkaliptosis is also a promising strategy in drug-resistant cancers like acute myeloid leukemia (AML), where alkaliptosis overcomes BCL-2-mediated resistance [11,44].

The inflammatory response caused by alkaliptotic damage is mediated through a high-mobility group box 1 (HMGB1) pathway. HMGB1 is released to the extracellular matrix after alkaliptosis and then binds to advanced glycosylation end product-specific receptor (AGER) in macrophages. This process activates the stimulator of the interferon response cGAMP interactor 1 (STING1) pathway, resulting in the production of pro-inflammatory cytokines [46]. Alkaliptosis is considered a promising mechanism not only in PDAC therapy but also in gastric cancer and acute myeloid leukemia treatment [19,44].

## 4. Oxeiptosis

Oxeiptosis is a term referring to the ROS-induced, caspase-independent, non-inflammatory apoptosis-like cell death pathway [13]. Increased levels of reactive oxygen species (ROS) are recognized contributors to cell proliferation and survival. However, when ROS reach pathologically elevated levels, they can induce oxidative stress, resulting in conformational changes in macromolecules such as proteins, lipids, and DNA. Oxidative stress can also lead to structural and functional abnormalities in cellular organelles, ultimately compromising cellular integrity and directing cells toward cell death via apoptosis [47].

Recently elucidated, oxeiptosis sheds light on a signaling pathway specifically associated with oxidative stress. Indeed, ROS are essential for many types of cell death (apoptosis, necroptosis, ferroptosis), mainly associated with inflammasomes and caspase-dependent pathways. Recently elucidated, oxeiptosis sheds light on a signaling pathway specifically associated with oxidative stress, but without triggering an inflammatory response. Unlike ferroptosis, which involves lipid peroxidation, or apoptosis, where ROS serve as secondary messengers in caspase-dependent pathways, oxeiptosis uniquely operates through the KEAP1–PGAM5–AIFM1 complex, selectively targeting oxidative stress-induced damage while maintaining cellular integrity [14,48]

To comprehend this intricate process, a detailed understanding of the key proteins modulating the oxeiptotic pathway is imperative. Noteworthy among these regulators are Kelch-like Ech-associated protein 1 (KEAP1), phosphoglycerate mutase family member 5 (PGAM5), and apoptosis-inducing factor mitochondrion-associated 1 (AIFM1) [47]. KEAP1, an endogenous inhibitor of NRF2 (nuclear factor erythroid-derived 2-like), serves as an oxidative stress sensor, assessing intracellular ROS levels. Under low oxidative stress, KEP1 undergoes a conformational change and dissociates from NRF2, a transcription factor that controls numerous expressions of cytoprotective antioxidant genes. KEAP1 interaction with PGAM5 is crucial for the regulation of mitochondrial dynamics and programmed cell death [49]. When confronted with elevated ROS concentrations, KEAP1 transitions from a cytoprotective state to a death-inducing mode by dissociating from PGAM5. Subsequently, PGAM5 is internalized into the mitochondrial lumen, where it dephosphorylates AIFM1—a substrate that facilitates DNA degradation—induces chromatin condensation, and orchestrates cell death. This finely tuned interplay among KEAP1, PGAM5, and AIFM1 highlights the intricacies of the oxeiptotic pathway, providing valuable insights into the regulation of oxidative stress-induced caspase-independent cell death [13,14,47].

Therefore, ROS levels, under the surveillance of KEAP1, exert regulatory influence over the constitution and modulate the composition of the KEAP1–PGAM5 complex. Elevated ROS concentrations culminate in the activation of the cytotoxic program. According to a study performed by Scaturro et al., oxeiptosis upon detrimental ROS levels regulates negatively inflammatory responses, serving as an additional cell death pathway [50]. Oxeiptosis is known to be induced by various stimuli such as H_2_O_2_ exposure, ozone exposure, viral infections, and the anticancer drug 5-fluorouracil, as well as by macrophages in neoplastic cells, and represents a promising target for the development of novel tumor suppression treatments [20].

**Figure 4:**
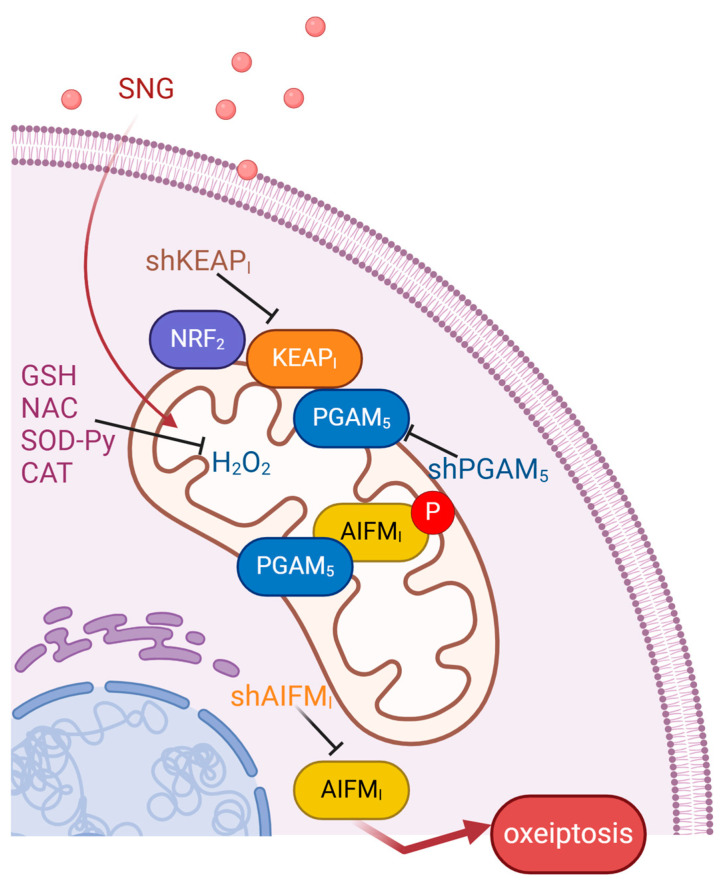
Schematic diagram of how SNG induces oxeiptosis in colorectal cancer cells based on [20]. SNG causes ROS accumulation in cancer cells, leading to activation of the KEAP1–PGAM5–AIFM1 signaling pathway. KEAP1 detects ROS and dissociates from NRF2, facilitating PGAM5 translocation to the mitochondria. There, PGAM5 dephosphorylates AIFM1, which causes chromatin condensation and DNA fragmentation, and mitochondrial dysfunction, hallmarks of the oxeiptosis phenotype. Oxeiptosis is characterized by ROS-induced regulated cell death with minimal inflammation, distinguishing it from other forms of regulated necrosis. SNG—sanguinarine, NRF2—nuclear factor erythroid-derived 2-like, KEAP1—Kelch-like Ech-associated protein 1, PGAM5—phosphoglycerate mutase family member 5, AIFM1—apoptosis-inducing factor mitochondrion-associated 1, GSH—glutathione, NAC—N-acetyl-cysteine, Sod-Py—sodium pyruvate, Cat—catalase.

Oxeiptosis, a recently characterized form of regulated cell death, can play a critical role in the pathogenesis of various diseases, including autoimmune disorders, neurodegenerative diseases, and inflammatory conditions [51]. Its mechanism, driven by reactive oxygen species (ROS) and involving the KEAP1–PGAM5–AIFM1 pathway, is important in terms of maintaining cellular homeostasis and responding to oxidative stress. Thus, understanding oxeiptosis would be crucial not only in disease progression but also as a potential therapeutic target. According to the research of Pllchankandy et al., sanguinarine (SNG, benzophenanthridine alkaloid) inhibits the growth of colorectal cancer (CRC) cells via H_2_O_2_-dependent activation of the KEAP1–PGAM5–AIFM1-induced oxeiptosis (Figure 4). Figure 4 highlights the role of SNG in inducing oxeiptosis by triggering ROS production and thus activation of KEAP1–PGAM5–AIFM1 signaling pathway. Those actions lead to chromatin condensation, DNA fragmentation, and mitochondrial dysfunction, which result in caspase-independent and non-inflammatory cell death. Colorectal cancer stands out as one of the most lethal cancers, characterized by high resistance to radiotherapy and chemotherapy coupled with an ability to evade apoptotic cell death. Consequently, the induction of oxeiptosis by natural products and their analogues, as demonstrated by SNG, emerges as an appealing avenue for exploration in the quest for innovative therapeutic strategies against CRC [20,49]. Furthermore, studies by Nasirzadeh et al. have provided results that alantolactone (ALT, sesquiterpene lactone) is a potential treatment option for ovarian cancer suppression through the induction of oxeiptosis. ALT reduces the levels of glutathione and inhibits thioredoxin reductase, which is followed by the accumulation of ROS in cancer cells. ROS overproduction leads to the activation of the oxeiptosis pathway, shedding new light on anticancer therapy strategies and allowing the limitation of compensatory side effects of traditional surgery [21].

## 5. Comparison and Contrast

Methuosis, alkaliptosis, and oxeiptosis represent distinct forms of regulated cell death (Table 2). Methuosis is one of the recent additions to the list of nonapoptotic cell death phenotypes. It is associated with the displacement of the cytoplasm by large fluid-filled vacuoles derived from macropinosomes [10]. Macropinocytosis is a clathrin-independent endocytic process by which cells internalize extracellular fluid, nutrients, and proteins in vesicles (macropinosomes) [25]. Methuosis is initiated by exorbitant stimuli that cause cytoplasmic absorption, leading to the formation of tiny vesicles that eventually combine to form large vacuoles. This process is linked to the disruption of macropinocytosis, an actin-driven mechanism activated by various stimuli. This disruption results in the enlargement of vesicles instead of the typical centripetal migration and fusion with lysosomes. The involvement of Rac1 and Arf6 in the process is crucial. The activation of Ras genes triggers methuosis by promoting macropinocytosis and vacuole formation. Cellular outcomes of methuosis involve impaired endocytosis, disrupted membrane trafficking, and cytoplasmic vacuolation, resulting in cell death. The process lacks the typical apoptotic features such as cell shrinkage, chromatin condensation, and plasma membrane blebbing and involves a nonapoptotic form of cell death [26,28,52].

Alkaliptosis, another form of regulated cell death distinct from apoptosis and necroptosis, involves cellular organelle swelling and plasma membrane rupture triggered by intracellular alkalization. Small-molecule compounds like JTC801 begin the initiation of the process and rely on major signaling pathways, including NF-κB-CA9 and ATP6V0D1-STAT3 [12,44]. Alkaliptosis involving changes in intracellular pH triggers cell death pathways and can be induced by compounds such as JTC801, offering potential benefits in alleviating symptoms of pain and anxiety in cancer patients [43]. Alkaliptosis can lead to cell cycle arrest or mitochondrial dysfunction [53,54].

Oxeiptosis is characterized by its reliance on intracellular reactive oxygen species (ROS) sensing (involves the engagement of KEAP1, with the mitochondria-tethered phosphatase PGAM5) and activation (elevated ROS levels activate the oxeiptotic pathway, influencing diverse biological functions and developmental stages). This process represents a non-inflammatory, caspase-independent pathway induced by oxidative stress, leading to damage to cellular components and cell death [13,14].

The regulation of methuosis, alkaliptosis, and oxeiptosis is intensely affected by the following factors: tumor microenvironment, oncogenic mutations, and cellular stressors. It was noted that in methuosis, oncogenic mutations like those in Ras genes could drive excessive micropinocytosis [26]. At the same time, the hypoxic and nutrient-depleted conditions of the tumor microenvironment may enhance macropinosome formation and vacuolization. Similarly, alkaliptosis can be modulated by intracellular pH dynamics, which are often altered by tumor-associated acidosis, leading to intracellular alkalization and activation of signaling pathways such as NF-κB-CA9 [11]. On the other hand, oxeiptosis is closely involved in oxidative stress levels, where ROS production is a symptom of both oncogenic signaling and cellular stressors within the tumor microenvironment. These conditions promote KEAP1 and PGAM5, modulating the susceptibility of cells to this non-inflammatory cell death pathway [49]. Methuosis, alkaliptosis, and oxeiptosis, while sharing some common features, exhibit distinct molecular mechanisms and cellular outcomes. Understanding these diverse forms of regulated cell death and how various factors intersect with and regulate these distinct pathways highlights their therapeutic potential and the need for precision approaches that account for tumor-specific contexts.

## 6. Exploration of the Therapeutic Potential of Methuosis, Alkaliptosis, and Oxeiptosis

Studies have shown that alkaliptosis, methuosis, and oxeiptosis may be useful tools in fighting cancer (Table 3). In this part, several studies will be presented. They focus on substances that showcase the ability to induce those processes and their applications in treating many forms of tumors, which affect many patients every year.

### 6.1. Methuosis in Cancer Therapy

With the discovery of methuosis, new potential cancer treatment options emerged. Several molecules that trigger this process have recently been found, and their potential in cancer therapy is currently being tested [26]. Among them is epimedokoreanin C (EKC), which is a prenylated flavonoid extracted from *Epimedium koreanum*. A study published in 2021 used EKC in human lung cancer NCI-H292, A549 cells, and bronchial epithelial 16HBE cells. The results showed that EKC inhibited the growth of lung cancer NCI-H292 and A549 cells by indicating a rapid and striking accumulation of numerous cytoplasmic vacuoles within 12 h. Additionally, EKC inhibited the migration of cancer cells. Moreover, the study showed that EKC could sensitize lung cancer cells to commonly used chemotherapy agents such as doxorubicin or etoposide. When it came to bronchial epithelial cells, EKC had no significant impact on them [36].

Another study worth mentioning shows the effects of using DZ-514 to induce methuosis in triple-negative breast cancer. In TNBC, metastasis is common, and it tends to be a rather aggressive type of cancer. Therefore, predictions rarely are optimistic. Treatment with DZ-514 may change this fact, since in this paper, this chemical compound was claimed to induce cytoplasmic vacuolization, known as methuosis (specifically through the ROS-MKK4-p38 pathway). Tumor cell viability and proliferation were successfully inhibited, both in cell lines and a mouse xenograft model [38].

### 6.2. Alkaliptosis in Cancer Therapy

Gastric cancer is undoubtedly one of the most common malignancies. Usually, it is diagnosed in late stages since the symptoms are not that noticeable, and screening is often irregular. Currently, various targeted therapies are available, but targeting alkaliptosis could enhance this problem [55]. Recent research demonstrated promising outcomes of using arenobufagin (ArBu) in combination with CDDP in the treatment of gastric cancer. In this experiment, the ASG andMKN-45 cell lines and mice with tumors were exposed to ArBu and CDDP. The researchers observed increased levels of IKBKB and NF-κB/p65 and decreased levels of CA9 in AGS and MKN-45 cells, which suggests activation of alkaliptosis. The growth of tumors and gastric cancer cell viability was inhibited. ArBu and CDDP used in mice decreased tumor growth, and the level of alkaliptosis-related proteins confirmed that it was due to alkaliptosis. In addition, no side effects in mice were observed [19].

Gastric cancer is not the only malignancy that might potentially be treated by alkaliptosis. It has been discovered that a molecule called JTC801 was a trigger that caused alkaliptosis in pancreatic cancer cells, prostate cancer cells, melanoma cells, kidney carcinoma cells, and central nervous system cancer cells. The efficacy of JTC801 was tested in vitro and in vivo [56]. Furthermore, the lack of toxic effects in healthy tissues in tested mice might mean that JTC801 only targets tumor cells [12].

### 6.3. Oxeiptosis in Cancer Therapy

Ionescu et al. noticed that sanguinarine (SNG) is effective in treating colorectal cancer, which is known to be one of the deadliest cancers. Although new methods for treatment have been developed, an increase in number of cases in people under 50 has been observed. SNG may be an efficient way of inducing oxeiptotic cell death, and using it in the treatment could improve prognosis [57]. It was shown that SNG induced in CRC cell lines increased the level of hydrogen peroxide, and consequently, SOD1 and SOD2 proved to be upregulated. Furthermore, it induced the KEAP1–PGAM5–AIFM1 pathway and led to cell death. The effects of sanguinarine were also tested in vivo. Tumor growth was inhibited after approximately 3 weeks of SNG treatment. Ser116 dephosphorylation in AIFM1 in tumor tissues indicated cell death via oxeiptosis. No drastic body mass change was observed in mice. Also, histopathological examination of mouse kidneys and livers did not reveal any abnormalities. In conclusion, SNG might be a new potential therapy option [20].

Yet another application of oxeiptosis may revolutionize the treatment of ovarian cancer, which is known to be the eighth-most common cancer among women and is often diagnosed in late stages. The treatment is a combination of chemotherapy and surgery. Current drugs that are being used to treat this type of tumor have many unpleasant side effects. Therefore, more options are being explored. One of them is the alantolactone (ALT) herbal drug, which has also shown anti-tumor potential in other types of cancers, such as lung, breast, or cervical cancer. In another study, the effects of ALT on the SKOV-3 ovarian cancer cell line were also investigated. The obtained results revealed that ALT increases the level of ROS by reducing the level of glutathione and thioredoxin reductase in cancer cells. This means that ALT inactivates the main antioxidant system in the cell, which results in oxidative stress. Additionally, ALT decreases transcription and translation of Nrf2 in SKOV-3. It leads to oxeiptosis; therefore, the proliferation and viability of cancer cells significantly decrease [21].

### 6.4. Challenges and Opportunities

Cell death mechanisms modulation plays a key role in the development of cancer. Many forms of cancer cells have evolved and gained resistance to many apoptosis-based mechanisms. Therefore, nonapoptotic pathways may put a new complexion on conventional cancer treatment [36]. These processes are a recent discovery, so more tests must be conducted to prove their efficacy in cancer treatment. No case studies have yet been published on using alkaliptosis, methuosis, and oxeiptosis in cancer therapy. The available studies were performed on cell culture or animal models and are shown in Table 3.

As was noted, one of the primary challenges is deciphering the heterogeneity of cell populations that undergo different types of cell death. The identification of distinct populations within a tumor that is susceptible to these nonapoptotic pathways requires advanced single-cell analysis techniques and robust biomarkers. The lack of reliable markers for nonapoptotic cell death poses a significant hurdle to translating these pathways into clinical applications. Another problem is multidrug resistance (MDR) and drug development that would effectively target these pathways without enhancing MDR effects [50]. Moreover, tumors are able to adapt to therapeutic pressure, which requires combination therapies or adaptive strategies to mitigate the MDR phenomenon [58]. While we are capable of studying apoptosis and necrosis, these nonapoptotic mechanisms, i.e., new types of cell death, remain a significant challenge to identify. Alkaliptosis, methuosis, and oxeiptosis open new ways to overcome MDR to conventional therapies. Targeting alternative cell death pathways may provide a counterpart to available treatments. As shown in Table 3, preliminary data suggest potential effectiveness against various cancer types, but further research is required to optimize drug delivery, dosing, and patient selection criteria. Thus, significant challenges remain, and the exploration of nonapoptotic cell death mechanisms represents a new direction in cancer treatment.

## 7. Conclusions

The previous sections explored a few forms of cell death, the history of their discovery, and their influence on neoplastic cells. Identifying those numerous and complicated pathways that drive cell death is critical for understanding illnesses and may aid in the development of novel therapeutics. Researching those non-canonical cell death mechanisms may lead to a paradigm shift in our apprehension of cancer biology, offering new dimensions for therapeutic exploration.

Thus far, scientists have concluded that the significance of methuosis lies in its distinctive engulfment of live cancer cells, providing a distinct target for intervention. Alkaliptosis, with its alkaline pH-induced cell death, represents an innovative strategy to exploit the tumor microenvironment for therapeutic gain. Oxeiptosis, a process intricately tied to oxidative stress, opens avenues for targeted therapies focusing on redox modulation.

As we have demonstrated here, the complex landscape of cancer therapy and the integration of these unconventional pathways into treatment strategies hold immense promise. The potential synergy between traditional modalities and these emerging mechanisms could enhance treatment efficacy while minimizing adverse effects. However, more research and clinical validation are needed. In the coming years, further investigations and clinical trials will be crucial in establishing the practical feasibility and safety of harnessing methuosis, alkaliptosis, and oxeiptosis in the clinical setting. Ultimately, this pursuit not only deepens our understanding of cancer biology but also holds the potential to redefine the therapeutic landscape, offering new possibilities to cancer patients.

## Figures and Tables

**Table 1:** Summary of compounds triggering methuosis, their effects, associated cell lines, and animal models.

Compound	Mechanism and Effect	Cell Line/ANIMAL Model	Ref.
Synthetic indole-based chalcones (MIPP, MOMIPP)	Overstimulate RAS genes and activate the JNK1/2 stress kinase pathway, resulting in the activation of PIKFYVE and leading to quick vacuolization	Glioblastoma xenograft model	[26]
Methamphetamine (METH)	Activates Rac1; induces MIPP-like macropinosomes without lysosome fusion	Neuroblastoma cells	[26]
Vacquinols—quinolone derivatives	Trigger accelerated cell death	Glioblastoma, hepatocellular carcinoma	[26]
AS1411—quadruplex-forming oligodeoxynucleotide that binds to nucleolin as an aptamer	Causes cell swelling and cytoplasmic vacuoles, i.e., hyperstimulation of macropinocytosis	DU145 prostate cancer cells and Hs27 non-malignant skin fibroblasts	[26,33]
F14512	Induces accumulation of multi-lamellar and vesicular bodies and increased β-galactosidase activity	A549 non-small-cell lung cancer cells	[26,34]
Silmitasertib precursor	Inhibits protein kinase CK2	Cholangiocarcinoma, colorectal cancer	[26]
CD99 monoclonal antibodies	Produce Rab5-positive endocytic vesicles through IGF-1R/RAS/Rac1 signaling	Ewing sarcoma	[26,35]
Ursolic acid derivative	Overactivates micropinocytosis, leading to cell death	HeLa cells–cervix adenocarcinoma	[26]
Epimedokoreanin C (EKC)—prenylated flavonoid from the herb *Epimedium koreanum*	Activates extreme Rac1 vacuolation and regulates Arf6 expression	Lung cancer cells	[36]
Spiropachysine A—primary active steroidal alkaloid from the herb *Pachysandra axillaries Franch*. *var. styiosa (Dunn) M. Cheng*	Stimulates methuosis through cytoplasmic vacuolization	Hepatocellular carcinoma (in vitro, in vivo)	[37]
DZ-514—N-phenyl-4-pyrimidinediamine derivative	Activates MKK4 pathway; inhibits tumor growth	Triple-negative breast cancer (TNBC), xenograft mouse model	[38]
Jaspine B (JB)-natural anhydrous sphingolipid derivative	Triggers cytoplasmic vacuolation	Lung adenocarcinoma cells with G12S K-Ras	[39]
DMBP—methyl 2,4-dihydroxy-3-(3-methyl-2-butenyl)-6-phenethylbenzoate	Targets vacuolar protein sorting-associated protein 41 homologues (VPS41)—a subunit of homotypic fusion and vacuole protein sorting (HOPS) complex—and re-strains fusion of late endosomes with lysosomes	Melanoma cells	[40]
Tubeimoside 2 (TBM-2)	Activates MKK4 pathway, suppresses tumor growth	Hepatocellular carcinoma, xenograft mouse model	[41]
L55	Interacts with PIKFYVE, surpasses MOMIPP anticancer potential	Breast adenocarcinoma, cervical carcinoma xenograft	[42]
JH530	Promising anti-tumor effect	Triple-negative breast cancer (TNBC in vitro, in vivo)	[15]

**Table 2:** Comparison of methuosis, alkaliptosis, oxeiptosis, and other cell death pathways (apoptosis, necrosis, ferroptosis, pyroptosis).

Feature	Methuosis	Alkaliptosis	Oxeiptosis	Apoptosis	Necrosis	Ferroptosis	Pyroptosis
Trigger	Oncogenic Ras activation, excessive macropinocytosis	Intracellular alkalization, compounds like JTC801	Elevated ROS levels, oxidative stress	DNA damage, cytotoxic agents	Severe cellular damage, trauma	Iron accumulation, lipid peroxidation	Infection, inflammasome activation
Key Mediators	Rac1, Arf6, macropinosome formation	NF-κB-CA9, ATP6V0D1-STAT3 pathways	KEAP1, PGAM5	Caspases (caspase 3 and 9), Bcl-2 family	Calcium influx, mitochondrial dysfunction	Iron, ACSL4, GPX4	Caspase 1, inflammasome complex
Morphological Features	Cytoplasmic vacuoles formed by enlarged macropinosomes	Organelle swelling, plasma membrane rupture	Non-inflammatory cell death, ROS-induced mitochondrial damage	Cell shrinkage, chromatin condensation, membrane blebbing	Cell swelling, membrane rupture	Membrane integrity loss, lipid peroxidation damage	Cell swelling, pore formation in plasma membrane
Inflammation	Non-inflammatory	Pro-inflammatory	Non-inflammatory	Non-inflammatory	Highly inflammatory	Non-inflammatory or weakly inflammatory	Highly inflammatory
Energy Dependency	Energy-dependent process	Energy-dependent	Energy-dependent	ATP-dependent	ATP-independent	ATP-dependent	ATP-dependent
Reversibility	Irreversible	Irreversible	Irreversible	Irreversible	Irreversible	Irreversible	Irreversible
Role in Pathology	Observed in cancer cells, linked to tumor growth and resistance	Associated with cancer, therapeutic potential	Associated with oxidative stress-related diseases	Cancer, neurodegenerative diseases	Ischemic injury, inflammation	Cancer, neurodegenerative diseases	Infectious diseases, inflammatory syndromes
Therapeutic Potential	Anti-tumor strategies targeting Ras, macropinocytosis	Modulating intracellular pH, NF-κB inhibitors	Targeting ROS levels, antioxidants	Apoptosis-inducing agents for cancer	Limited therapeutic potential, focuses on prevention	Iron chelators, lipid peroxidation inhibitors	Inflammasome inhibitors

**Table 3:** The use of methuosis, alkaliptosis, and oxeiptosis in cancer therapy.

Molecule	Type of Cell Death	Cancer Cells Treated	Ref.
epimedokoreanin C (EKC)	methuosis	human lung cancer NCI-H292, A549 cell sbronchial epithelial 16HBE cells	[36]
DZ-514	methuosis	triple-negative breast cancer cells	[38]
arenobufagin (ArBu)	alkaliptosis	gastric cancer cells	[19]
JTC801	alkaliptosis	pancreatic cancer cells, prostate cancer cells, melanoma cells, kidney carcinoma cells, central nervous system cancer cells	[12]
sanguinarine (SNG)	oxeiptosis	CRC cell lines	[20]
alantolactone (ALT)	oxeiptosis	SKOV-3 ovarian cancer cell line	[21]

## Data Availability

Not applicable.
